# Preliminary report: Reduced hand sensory and motor function in persons living with heart failure

**DOI:** 10.1371/journal.pone.0312468

**Published:** 2024-11-15

**Authors:** Hidetaka Hibino, Stacey L. Gorniak

**Affiliations:** 1 Department of Physical Therapy, Movement, and Rehabilitation Sciences, Northeastern University, Boston, Massachusetts, United States of America; 2 Department of Health and Human Performance, University of Houston, Houston, Texas, United States of America; Aalto University School of Science and Technology: Aalto-yliopisto Insinooritieteiden korkeakoulu, FINLAND

## Abstract

Despite the growing evidence highlighting reduced functional independence in persons living with heart failure (PwHF), the underlying mechanisms that lead to reduced functional independence in this patient population are unknown. Given the association between functional independence and fine motor skills, which are functionally related to hand sensory and motor functions, we hypothesized that PwHF exhibit reduced sensory and motor function of hands compared to healthy individuals. We recruited a total of 10 PwHF (age: 57.6 ± 12.5 years old, four females) and a total of age- & sex-matched healthy control individuals (age: 58.2 ± 12.2 years old, four females). Participants performed a wide range of tests assessing the level of independence, fitness, cognitive function, and hand sensorimotor function. While the level of independence was comparable between two groups, PwHF exhibited reduced sensory and motor function. Compared to healthy participants, the ability to identify an object via tactile and proprioceptive inputs was reduced in PwHF, though the tactile mechanoreceptor function showed normal integrity. Similarly, PwHF exhibited a decline in manipulating small objects and steady grip force production. Heart failure seems to have repercussions that extend to the sensorimotor control of hand actions in advance to a decline in functional independence. These results underscore the need of further investigation as to the underlying mechanisms of reduced sensorimotor function, potential intervention targets, and determine whether assessments of hand sensorimotor function can serve as a vehicle to quantify restoration of self-care functionality.

## Introduction

Approximately 6 million Americans 20 years of age or older live with heart failure, and an additional 2 million American adults are expected to develop heart failure in less than a decade [1, 2]. Heart failure is a complex syndrome characterized by structural or functional defects of ventricular filling or ejection fraction of the heart that do not meet metabolic requirements [3]. While dyspnea, fatigue, and fluid retention are symptoms commonly reported by persons living with heart failure (PwHF), indispensable reports demonstrating declined independence in activities of daily living (ADLs) are also mounting [4]. The risk of reduced independence in PwHF is 50% higher than healthy individuals [5]. The prevalence of reduced independence ranges from 11%–75% [4]. The dependence in PwHF seems to be multifactorial, such as severity, task difficulty, sex, age, and cognitive function [6–11].

Independence is associated with hand function, particularly fine motor skills [12]. Fine motor skills have been functionally associated with a sensory function and motor function of hands. For instance, fine motor skills decline as tactile spatial acuity is reduced, which highlights the significance of afferent feedback in object manipulation [13, 14]. Similarly, reduced fine motor skills and reduced brail-like pattern recognition, in which requires high-order cognitive function, coincide [15, 16]. The functional significance of pinch force steadiness for fine motor skills has also been demonstrated [17, 18].

Today, no comprehensive studies testing the potential causes responsible for reduced functional independence in PwHF exist. Given the link between independence and fine motor skills, which are functionally associated with the sensory and motor function of the hands, it is possible that these functions are affected by heart failure. To test these hypotheses, we assessed sensory and motor functions of hands in PwHF and age- & sex-matched healthy control individuals. We expected reduced self-reported functional independence in PwHF (Hypothesis #1), which are in line with other epidemiological evidence [10]. We expected reduced fine motor skills in PwHF, which may be a plausible cause for reduced independence in PwHF (Hypothesis #2). We expected heart failure-related decline in hand sensory function, assessed by tactile registration and an object recognition ability, and the pinch force steadiness (Hypothesis #3). Table 1 shows abbreviations that are used in this manuscript.

**Table 1:** Nonstandard abbreviations and acronyms.

ADL	activities of daily living
ALM	automatic linear modeling
ApEn	approximate entropy
BMI	body mass index
CO	control group
CV	coefficient of variation
DBP	diastolic blood pressure
HbA_1c_	glycated hemoglobin
HDL	high-density lipoprotein
IADL	instrumental activities of daily living
HFmrEF	heart failure with mid-range ejection fraction
HFpEF	heart failure with preserved ejection fraction
HFrEF	heart failure with reduced ejection fraction
LDL	low-density lipoprotein
MoCA	Montreal Cognitive Assessment
PCPST	pattern comparison processing speed test
PwHF	persons living with heart failure
RMSE	root mean square error
SBP	systolic blood pressure
SpO2	blood oxygen saturation
T2DM	type II diabetes mellitus
TC	total cholesterol

## Methods

### Participants

A total of 10 self-reported right-handed persons living with heart failure (PwHF, age: 57.6 ± 12.5 years old, four females) and a total of 10 self-reported right -handed age- & sex-matched healthy control participants (CO, age: 58.2 ± 12.2 years old, four females) were recruited from the Greater Houston area. All PwHF declared a diagnosis of heart failure. The hand dominance was determined by Edinburgh Handedness Inventory [19]. Participants who met the following criteria were excluded from the study: (1) taking medications that lead to sensorimotor dysfunction, (2) co-morbidities that affect sensorimotor function. The University of Houston Institutional Review Board approved the study protocol (IRB #: STUDY00002306). The study was conducted in accordance with the Declaration of Helsinki. A written informed consent form was obtained from all participants. The beginning and end of subject recruitment were June 1^st^, 2021 and September 30^th^, 2022, respectively.

### Experimental apparatus

#### Manipulandum

**Fig 1:**
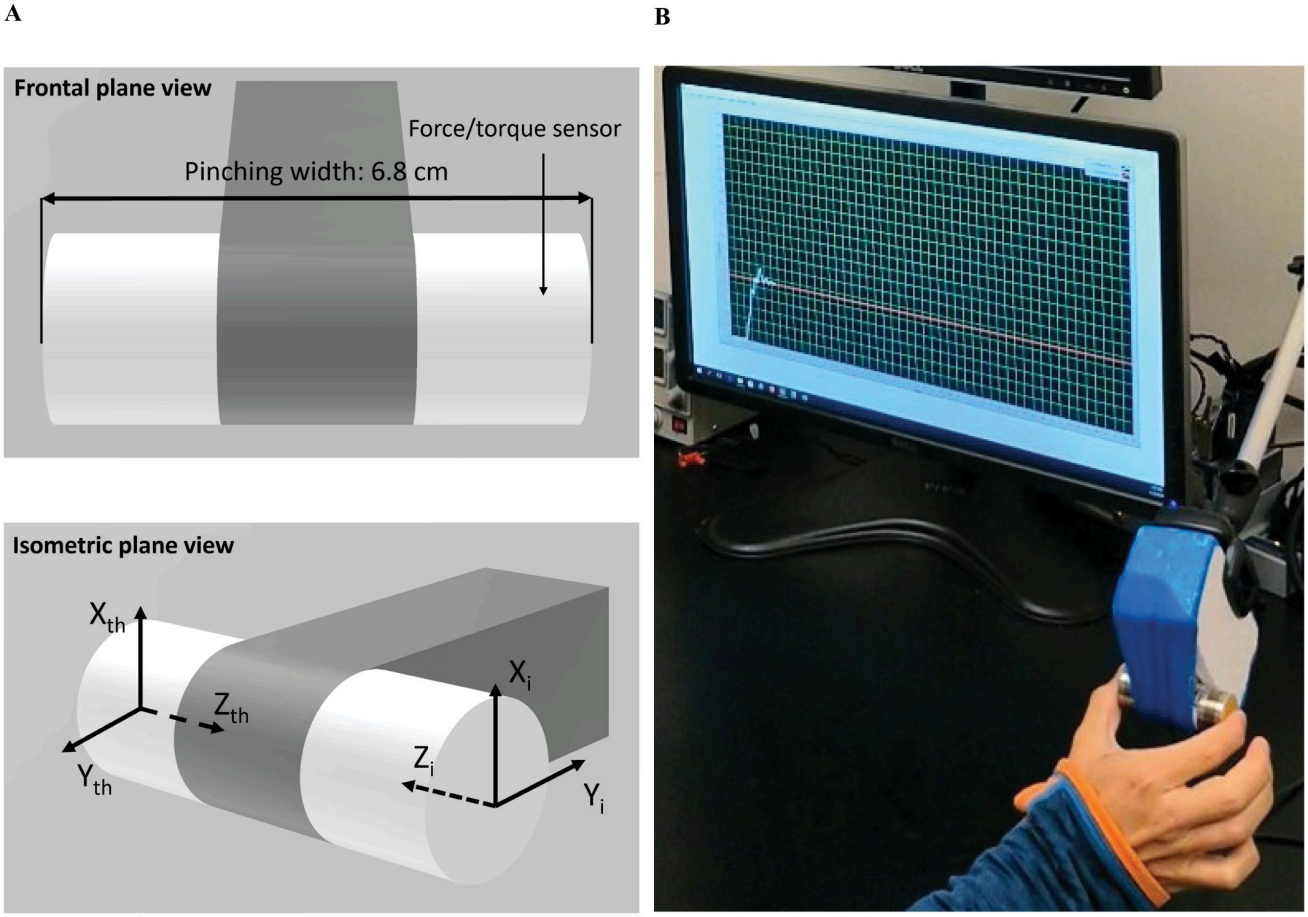
Diagrams of manipulandum. **A)** The manipulandum consisted of two identical force/torque transducers (shown in white). Local axes of the thumb and index sensors (X_th_, Y_th_, Z_th_ and X_i_, Y_i_, Z_i_; respectively) are shown. **B)** Individual interacting with the manipulandum during the constant pinch force tracking test.

A manipulandum was built to evaluate the ability to maintain submaximal pinch force (Fig 1). The manipulandum consisted of two identical six-component force-moment transducers (nano25 Transducers: ATI Industrial Automation, Garner, NC, USA) to record pinch force (aka: normal force) exerted by the digits one and two of the right hand. The distance between two contact surfaces for the two fingers was 6.8 cm. The manipulandum was firmly attached to a height-adjustable arm.

### Data collection

#### List of assessments

Assessments that were performed in this study are listed in Table 2. The table shows the function the assessment is evaluating, and the aspect of function that is assessed.

**Table 2:** List of assessments.

Name of assessments	The function the assessment is evaluating	The aspect of function
Katz ADL	Independence in basic daily task	Functional independence
Lawton IADL	Independence in complex daily task	Functional independence
Pattern Comparison Processing Speed Test	Processing Speed	Cognitive function
Grip and pinch force test	Hand muscle strength	Motor function
Six-minute walk test	Exercise intolerance	Motor function
Semmes-Weinstein monofilament test	Tactile sensitivity	Sensory function
Nine-hole peg test	Fine motor skills	Motor function
Stereognosis	Ability to process and integrate proprioception and tactile information	Sensory function
Constant pinch force tracking test	Ability to maintain submaximal pinch force	Motor function

#### Subject demographic and clinical characteristics

The demographic characteristics of participants, including age, sex, ethnicity, the existence of peripheral neuropathy, heart failure duration, and ejection fraction were collected via a self-reported questionnaire. The subtype of heart failure was further determined based on the value of self-reported ejection fraction. Weight and height were measured to quantify body mass index (BMI). Systolic (SBP) and diastolic (DBP) blood pressures, and blood oxygen saturation (SpO2) were collected via 10 Series Blood Pressure Monitor (Omron Healthcare, Inc., Bannockburn, IL, USA) and O2Ring (Viatom Technology Co., Arcadia, CA, USA), respectively. A1CNow+ (pts Diagnostics, Whitestow, IN, USA) was performed to assess glycated hemoglobin (HbA_1c_), while CardioCheck (pts Diagnostics) was used to assess total cholesterol (TC), high-density lipoprotein, (HDL), and low-density lipoprotein (LDL). Montreal Cognitive Assessment (MoCA) was delivered to evaluate general cognitive function. BMI, SBP, DBP, SpO2, HbA_1c_, TC, HDL, LDL, the total score of MoCA, and heart failure duration were used as health-related covariates in our statistical analysis.

#### Level of independence in daily living

The level of independence in daily living was determined through two self-reported questionnaires: (1) Katz index of independence in activities of daily living (ADLs), and (2) Lawton instrumental activities of daily living scale (IADLs). The Katz index of independence in ADLs determines the level of independence in six basic daily activities that are fundamental for living, while the Lawton IADLs assesses independence in more complex ADLs necessary for living [20, 21]; higher scores indicate independence in basic and complex ADLs.

#### Clinical cognitive function assessment

As processing sensory information is a critical function in controlling body, we measured visual information processing speed via NIH Toolbox Pattern Comparison Processing Speed Test (PCPST), which has a strong correlation with measurement assessing coding in stroke patient, via iPad [13, 22, 23]. Employing a cognitive task that axons of sensory neurons do not involve was critical, as PwHF may have neuropathy. During the test, the iPad displayed two images at the same time. The two images could be mismatched by color, number of items, or missing/extra parts. Participants were instructed to answer whether two images are same or not as quickly as accurately as possible by tapping “yes” or “no” button with their right hand. Practice sessions were given to become familiar with the task. The responses were recorded on iPad; scores of Pattern Comparison Processing Speed Test, which reflected the number of correct items completed in 90 seconds, was calculated by NIH Toolbox application. The fully demographically adjusted T-score, in which adjusts age, gender, race, ethnicity, and education, was used for data analysis [24]. Briefly, the T-score ranges from zero (0) to 100 and has a mean of 50 and a standard deviation of 10. The T-scores below 50 indicate that the processing speed is slower compared to age-, education-, sex-, and race/ethnicity-matched peers.

#### Clinical exercise assessment

Grip and pinch force test was delivered to evaluate maximum grip and pinch forces, respectively. The maximum grip and pinch forces, measures that are associated with cardiovascular mortality, of the right hand were recorded using a digital dynamometer and pinchmeter (Biometrics Ltd, Newport, UK), respectively [25, 26]. Details regarding this test can be found in a paper from our group [27]. The highest value across three trials was recorded as their maximal grip force and maximal pinch force [28].

The six-minute walk test was delivered to evaluate exercise intolerance, which is associated with functional status in persons living with heart failure and is related to peak oxygen consumption in cardiopulmonary exercise test [29, 30]. Participants were instructed to walk as far as possible for six minutes by walking in a hallway, in which they try to cover a distance as far as possible in six minutes. During the test the participants were permitted to slow down, to stop, and to rest as necessary. The timer started when the participants started walking. The participants were encouraged every minute by two examiners, who were following from behind as spotters. One examiner was holding a measuring wheel to record the total walking distance. At the sixth minute, the participants were instructed to stop walking; the total distance (m) displayed on the measuring wheel was recorded and used for data analysis.

#### Clinical hand sensorimotor assessment

Clinical hand sensory and motor functions were evaluated using Semmes-Weinstein monofilament test and nine-hole peg test, respectively. The Semmes-Weinstein monofilament test is a non-invasive sensory test commonly used to evaluate the threshold of tactile registration; it has been used to assess hand sensory function in different populations, such as persons living with type II diabetes and healthy older adults [31, 32]. The threshold of tactile registration was evaluated on the distal pad of the digits 1, 2, 5, hypothenar eminence, and 1^st^ dorsal interosseus of both hands. Each testing site was touched by a flexible filament that bent when pressure applied to the skin exceeds certain pressure [33]. The pressures applied to the testing site ranged between 0.07 g and 300 g. Participants were instructed to respond when they registered a stimulus on the testing sites. The threshold of tactile registrations was determined in a descending manner, such that the stimulation pressure declined as the participants successfully registered the stimulus three consecutive times. The smallest pressure (g) the participants successfully recognized three consecutive times was recorded as the threshold of tactile registration. The recorded threshold was log-transformed (log_10_(pressure(g))) for data analysis due to its nature of non-linearity [32].

The nine-hole peg test is a timed clinical test that evaluates fine motor skills [34]. The participants were instructed to complete the task as quickly as possible using only one hand. The time (seconds) from when the participants touched the first peg to when the participants placed the last peg in the container was recorded and was analyzed. Both hands were tested, starting with the right hand.

#### Stereognosis

**Fig 2:**
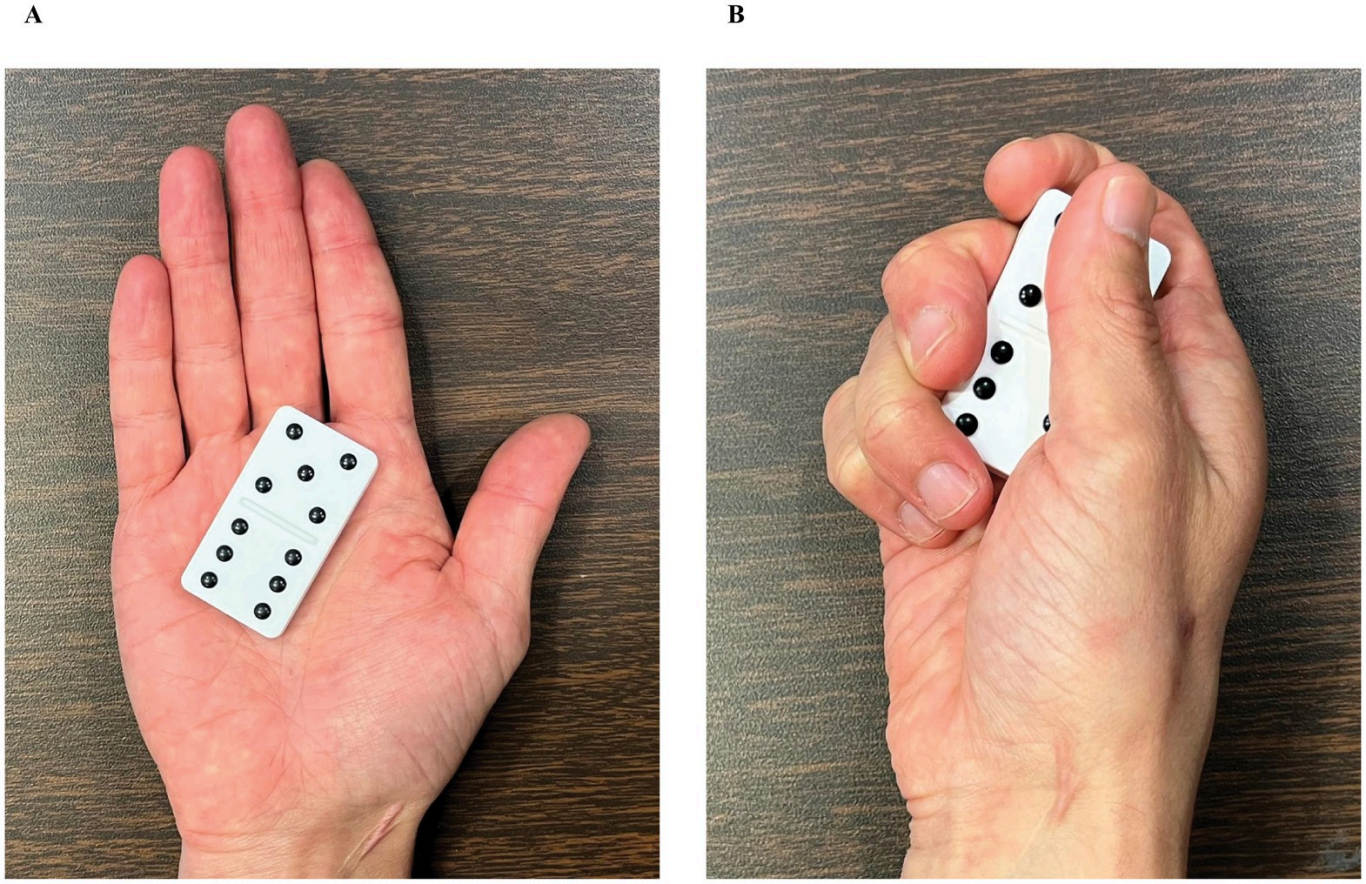
Image of dominos for stereognosis. Domino with raised dots for stereognosis task.

To test stereognosis, the ability to understand a three-dimensional object via integration of tactile and proprioceptive inputs, dominoes with raised dots were used (Fig 2) [35]. Similar tests asking participants to recognize objects or patterns of raised pins only with hand sensory information is administer in older adults and patient population, in which depicted hand sensory function impairment that was not captured by a clinical sensory test (e.g. Semmes-Weinstein Monofilament test) [15, 36, 37]. The dominoes with zero (0) or one (1) dot were not used in the study since they lack spatial orientation of dots. Additionally, no dominoes had two same numbers of dots. Furthermore, no dominoes were used twice in the test. As a result, there were a total of 10 different combinations that were available for stereognosis. Thus, a total of 10 dominoes (five dominoes for each hand) were used in the study.

Participants were instructed to close their eyes while one hand was supinated and was rested on a table. An experimenter placed a domino into the palm; the domino was oriented such that it was parallel to the digit 1. Once the experimenter said “go”, the participants started exploring the domino with the hand holding the domino. The response for each pattern of dots and the total time to answer both patterns on the domino were recorded. There were five trials for each hand. Both hands were tested; the order of testing hand was counterbalanced between participants. Response accuracy and response time of each trial was calculated by the accuracy of response for each pattern and the time from the auditory cue “go” until when the participants reported the last pattern of dots on the domino. The average of response accuracy and response time across five trials were quantified for both right and left hands.

To take the speed-accuracy trade-off into account, balanced integration score was quantified [38]. The balanced integration score is the difference between the standardized mean response accuracy and the standardized mean response time. The balanced integration score was calculated using following equation: *balanced integration score* = *ZRA*–*ZRT*, where ZRA and ZRT are the standardized mean response accuracy and the standardized mean response time, respectively. The standardized values were calculated as follows: $Zx=\frac{x-\;\mu x}{SDx}$, where x represents the interested variable (i.e. response accuracy and response time) and μ and SD are mean and standard deviation of the response accuracy and response time across both hands, respectively.

#### Laboratory-based hand motor assessment

Constant pinch force tracking test is widely used as an assessment of hand motor performance [17, 27, 39, 40]. Participants were instructed to produce a constant level of pinch force using digits 1 and 2 of their right hand on the manipulandum. The participants sat in front of a computer monitor that was placed on a table. The participants were instructed to match their pinch force to a target force presented on the computer monitor as accurately as possible. Two target forces were used in this test: 5% and 20% of their maximal pinch force. The target force and the produced pinch force were displayed visually via the computer monitor. There was a total of six trials (three trials for each target force). Each trial lasted 50 seconds, and one-minute rest interval was given after each trial. The order of target force was counterbalanced between participants.

### Data processing and analyses

Constant pinch force tracking test: The transducer signals were amplified and multiplexed using ATI hardware before routed to analog-to-digital (via two NI-9205 input modules and one cDAQ-9174 chassis: National Instruments, Austin, TX, USA). Using a customized LabVIEW (National Instruments) program, the force applied to each transducer on z-axis acquired at 100 Hz. The signals were processed with a customized MATLAB (Natick, MA, USA) program. The signals were low-pass filtered at 10 Hz using a 2^nd^ order zero-lag Butterworth filter [27]. Pinch force was defined as the sum of normal force applied to each transducer with thumb and index finger (Z_i_ + Z_t_). Based on a visual inspection of the signals, the first and last 10 seconds were removed as pinch force was not stabilized at the beginning of the trial and several participants failed to maintain at the target force until the trial end, respectively. One trial out of a total of 120 trials across conditions and participants were excluded from the data analyses as pinch force dropped to zero (0) in the middle of the task.

To evaluate the ability to produce constant pinch force over the time and the accuracy of pinch force, coefficient of variation (CV) and root mean square error (RMSE) of pinch force were calculated, respectively. Additionally, the approximate entropy (ApEn) of pinch force was quantified for better understanding in the regularity of pinch force production [41]. The ApEn value ranges between zero (0) and two; zero and two indicate the signal has the maximum regularity and maximum randomness, respectively. The regularity of pinch force production was assessed as a functional measurement in a wide range of patient population [27, 42]. The ApEn was quantified using MATLAB function *approximateEntropy*. The average CV, RMSE, and ApEn across the three trials of each target force was quantified for each subject and was analyzed.

### Statistical analysis

SPSS version 27 (IBM, New York, NY, USA) was used to perform statistical analyses. All significance levels were set at p < 0.05 before any statistical analysis, unless otherwise described. The data are shown as estimated mean ± standard error based on analysis of covariance (ANCOVA) otherwise stated.

The assumption of normal distribution was tested by measuring the skewness and kurtosis of the value in each level of combination and was met when the absolute value of skewness and kurtosis was less than |2| and |9|, respectively. Non-parametric statistical analyses were performed when the assumption of normal distribution was violated.

Independent t-tests were performed for all health-related covariates: BMI, SBP, DBP, SpO2, HbA_1c_, TC, HDL, LDL, the total score of MoCA. Between-subject analysis of variances (BS-ANOVAs) with *Group* (CO and HF) as a between-subject factor were performed for: ADLs, IADLs, maximal grip force, maximal pinch force, six-minute walk test, and Pattern Comparison Processing Speed Test. Two-way mixed design ANOVAs (MD-ANOVAs) were performed for: nine-hole peg test, response accuracy, response time, balanced integration score, CV, RMSE, and ApEn. While the between-subject factor for two-way MD-ANOVA was *Group*, the within-subject factors depended on the measurement: *Hand* (Right and Left) for nine-hole peg test, response accuracy, response time, and balanced integration score; *Target* (5% and 20%) for CV, RMSE, and ApEn. A three-way MD-ANOVA was performed for SMWT with *Group* as a between-subject factor, *and Hand* and *Site* (Palmar pad of digit 1, digit 2, digit 5, hypothenar eminence, and 1^st^ dorsal interosseus) as within-subject factors. The assumption of multi-sample sphericity was tested with Box’s M test; Mauchly’s test of sphericity was used to test the assumption of sphericity. The Huynh-Feldt correction was used if the sphericity was violated. If a statistically significant interaction was found in the MD-ANOVA, a Bonferroni correction was applied for follow-up analyses.

To examine whether any health-related covariates influenced the results of our statistical ANCOVA was performed for all variables. The covariates for each variable were determined via Automatic Linear Modeling (ALM) forward stepwise selection. For further understanding in the degree of linear association between variables and covariates, Person correlations were performed after ANCOVA. The effect size is reported in Cohen’s d for t-tests and Mann-Whitney U test, whereas partial eta-squared (η^2^) is reported for ANOVAs and ANCOVAs.

## Results

### Participants

Demographic and clinical characteristics of PwHF are shown in Tables 3 and 4. Information about the medication is reported in S1 Table. Half of PwHF reported their ejection fraction value. The type of heart failure was determined based on the self-reported ejection fraction value [3, 43]. Two PwHF reported the diagnosis of idiopathic peripheral neuropathy. Fingerstick blood test failed in one person living with heart failure, while the cholesterol test system failed to report the LDL value in four persons living with heart failure. Since 25% of the participants did not have the LDL value, the LDL value was excluded from statistical analysis. Independent t-tests for health-related covariates found a significant group difference of 2.4 for the total score of MoCA (t_18_ = 2.36, p < 0.05, Cohen d = 1.05). All other health-related covariates were comparable between groups.

**Table 3:** Demographic characteristics of PwHF.

Participant	Age (years)	Sex	Ethnicity	Heart Failure Duration (months)	Ejection Fraction (%)	Heart Failure type
1	67	Male	African American/Black	144	—	—
2	56	Female	African American/Black	208	45	HFmrEF
3	69	Male	Caucasian/White	84	45	HFmrEF
4	71	Male	Caucasian/White	130	—	—
5	53	Male	African American/Black	45	—	—
6	50	Male	African American/Black	7	57	HFpEF
7	51	Female	Caucasian/White	77	—	—
8	44	Male	Hispanic/Latino	76	—	—
9	78	Female	Caucasian/White	1	68	HFpEF
10	43	Female	African American/Black	41	40	HFrEF
	Mean ± SD	Frequency	Frequency	Range	Range	Frequency
PwHF n = 10	57.6 ± 12.5	Male n = 6 Female n = 4	AA/B n = 5 C/W n = 4 H/L n = 1	1–208	45–68	HFmrEF n = 2 HFpEF n = 2 HFrEF n = 1
CO n = 10	58.2 ± 12.2	Male n = 6 Female n = 4	AA/B n = 1 C/W n = 6 H/L n = 1 M n = 1 O n = 1	NA	NA	NA

*AA/B*, African American/Black; *C/W*, Caucasian/White; *CO*, Control group; *H/L*, Hispanic/Latino; *HF*, heart failure group; *HFmrEF*, heart failure with mid-range ejection fraction; *HFpEF*, heart failure with preserved ejection fraction; *HFrEF*, heart failure with reduced ejection fraction’ *M*, Multiracial; *NA*, not applicable; *O*, other;—denotes unknown HF type or ejection fraction value

**Table 4:** Clinical characteristics of persons living with heart failure.

Participant	BMI (kg/m^2^)	Systolic (mmHg)	Diastolic (mmHg)	HbA_1c_ (%)	TC (mg/dL)	HDL (mg/dL)	LDL (mg/dL)	SpO2 (%)	MoCA Score
1	20.7	158	89	—	—	—	—	98	27
2	31.9	115	76	6.2	175	62	—	98	25
3	22.7	141	85	4.8	100	54	—	99	23
4	31.5	114	68	5.7	133	38	79	99	28
5	24.4	158	122	5.6	126	36	65	98	21
6	32.6	150	82	5	206	39	127	97	28
7	43.2	136	86	7.9	312	49	High LDL	98	22
8	27.3	123	64	4.7	149	75	63	98	26
9	30.5	123	75	5.9	147	52	76	98	27
10	34.6	194	103	5.8	100	34	—	98	24
	Mean ± SD	Mean ± SD	Mean ± SD	Mean ± SD	Mean ± SD	Mean ± SD	Mean ± SD	Mean ± SD	Mean ± SD
PwHF	29.9 ± 6.6	141.2 ± 24.8	85.0 ± 17.1	5.7 ± 1.0	160.9 ± 66.0	48.8 ± 13.7	82.0 ± 26.1	98.1 ± 0.6	25.1 ± 2.5*
CO	30.5 ± 4.0	132.4 ± 21.4	80.3 ± 10.9	5.4 ± 0.5	178.8 ± 33.4	49.0 ± 14.5	100.3 ± 34.4	97.0 ± 1.6	27.5 ± 2.0

*BMI*, body mass index; *CO*, control group; *HbA*_*1c*_, glycated hemoglobin; *HDL*, high-density lipoprotein; *HF*, heart failure group; *LDL*, low-density lipoprotein; *MoCA*, Montreal Cognitive Assessment; *SD*, standard deviation; *SpO2*, blood oxygen saturation; *TC*, total cholesterol;—denotes data collection failure; *, p < 0.05

### Level of independence

Self-reported independence was comparable between CO and PwHF, suggesting that PwHF did not perceive any declines in self-care functionality, as indicated by Mann-Whitney U test. Comparable independence scores between CO and HF were found (ADLs: *U* = 45.0, *z* = -1.0, p = 0.32, Cohen’s d = -0.45, IADLs: *U* = 45.0, *z* = -1.0, p = 0.32, Cohen’s d = -0.45). ALM did not identify any covariates for ADLs and IADL data.

### Cognitive function and exercise capacity

**Fig 3:**
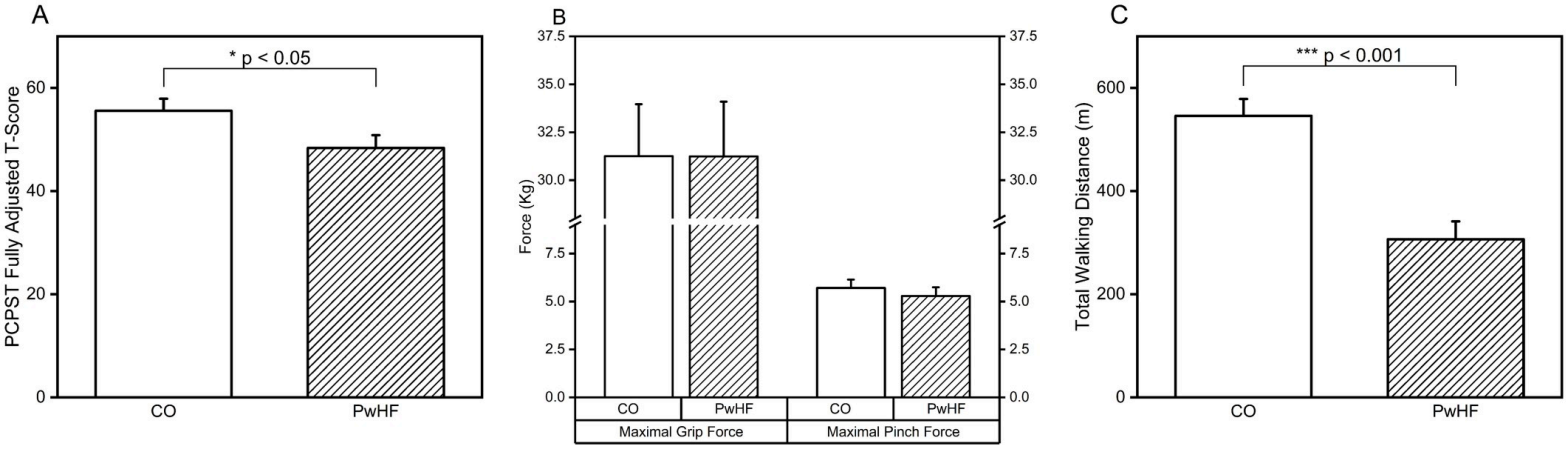
Cognitive function and exercise capacity data. **A**: Fully-adjusted Pattern Comparison Processing Speed Test (PCPST) for both groups. **B**: Maximal grip force (left) and maximal pinch force (right) for both groups. **C**: The distance covered in six-minute walk test for both groups.

Speed of information processing was slower in PwHF compared to CO; the group difference remained significant while controlling for covariates. Independent t-test revealed a statistically significant group difference of 8.2 (t_18_ = 2.25, p < 0.05, Cohen’s d = 1.01) between CO (Adjusted score: 55.6 ± 10.2) and PwHF (Adjusted score: 47.4 ± 5.3) in PCPST. ALM identified HDL as a covariate and BS-ANCOVA was performed. The *Group* effect remained significant (F_1,16_ = 4.48, p < 0.05, η^2^ = 0.22) while controlling HDL (Fig 3A).

Measurements of hand strength did not differ between CO and PwHF. Independent t-tests found no group differences in either maximal grip force and maximal pinch force (maximal grip force: t_18_ = 0.63, p = 0.54, Cohen’s d = 0.28, maximal pinch force: t_18_ = 0.63, p = 0.54, Cohen’s d = 0.28). ALM identified HbA_1c_ as a covariate for maximal grip force, while HbA_1c_ and SBP were identified as the covariates for maximal pinch force; BS-ANCOVAs with *Group* were performed for both maximal grip force and maximal pinch force. BS-ANCOVAs demonstrated that both maximal grip force and maximal pinch force were comparable between CO and PwHF while controlling for covariates (Fig 3B). HbA_1c_ was not significantly associated with maximal grip force, while HbA_1c_ and SBP were both significantly associated with maximal pinch force (HbA_1c_: F_1,15_ = 7.05, p < 0.05, with r_19_ = -0.49, p < 0.05; SBP: F_1,15_ = 5.93, p < 0.05, with r_20_ = 0.39, p = 0.09).

PwHF exhibited greater exercise intolerance compared to CO; the difference between groups remained significant after controlling covariates. Independent t-test indicated that walking distance was longer in CO (509.1 ± 107.6m) compared to HF (367.0 ±138.3m), a statistically significant group difference of 142.1m (t_18_ = 2.56, p < 0.05, Cohen’s d = 1.15). ALM identified TC and SpO2 as covariates. BS-ANCOVA found a significant *Group* difference that persisted in the total walking distance (F_1,15_ = 22.22, p < 0.001, η^2^ = 0.60), while controlling TC and SpO2 (Fig 3C). Both covariates were significantly associated with six-minute walk test (TC: F_1,15_ = 7.02, p < 0.05, with r_19_ = -0.23, p = 0.34; and SpO2: F_1,15_ = 6.49, p < 0.05 with, r_20_ = 0.05, p = 0.83).

### Hand sensory function

Threshold of tactile sensitivity was comparable between CO and PwHF, while the right hand had higher tactile threshold compared to the left hand across groups. The tactile threshold depended on the testing site as well. When covariates were controlled, only the main effect of *Site* remained significant. Hypothenar eminence and 1^st^ dorsal interosseus exhibited lower tactile sensitivity compared to the distal pad of digits 1, 2, and 5. Three-way MD-ANOVA found significant main effects of *Hand* (F_1,18_ = 5.64, p < 0.05, η^2^ = 0.24) and *Site* (F_1,18_ = 3.65, p < 0.01, η^2^ = 0.17). Pairwise comparison of *Hand* revealed that the right hand had a better tactile sensitivity compared to the left hand (right hand vs. left hand (mean ± standard error (SE)): -0.34 ± 0.09 vs. -0.48 ± 0.08). Pairwise comparison of *Site* found lower tactile sensitivity in the hypothenar eminence had a higher tactile threshold compared to the digits (digit 1 vs. hypothenar eminence (mean ± SE): -0.47 ± 0.10 vs. -0.30 ± 0.95, digit 2 vs. hypothenar eminence (mean ± SE): -0.48 ± 0.09 vs. -0.30 ±.95, digit 5 vs. hypothenar eminence (mean ± SE): -0.54 ± 0.10 vs-0.30 ± 0.95). Pairwise comparison of *Site* revealed that 1^st^ dorsal interosseus (radial nerve) had higher tactile threshold compared to the distal pads of digits (digit 1 vs. 1^st^ dorsal interosseus (mean ± SE): -0.47 ± 0.10 vs. -0.27 ± 0.12, digit 2 vs. 1^st^ dorsal interosseus (mean ± SE): -0.48 ± 0.09 vs. -0.27 ± 0.12, digit 5 vs. 1^st^ dorsal interosseus (mean ± SE): -0.54 ± 0.10 vs. -0.27 ± 0.12). There was no difference between the hypothenar eminence and 1^st^ dorsal interosseus.

**Fig 4:**
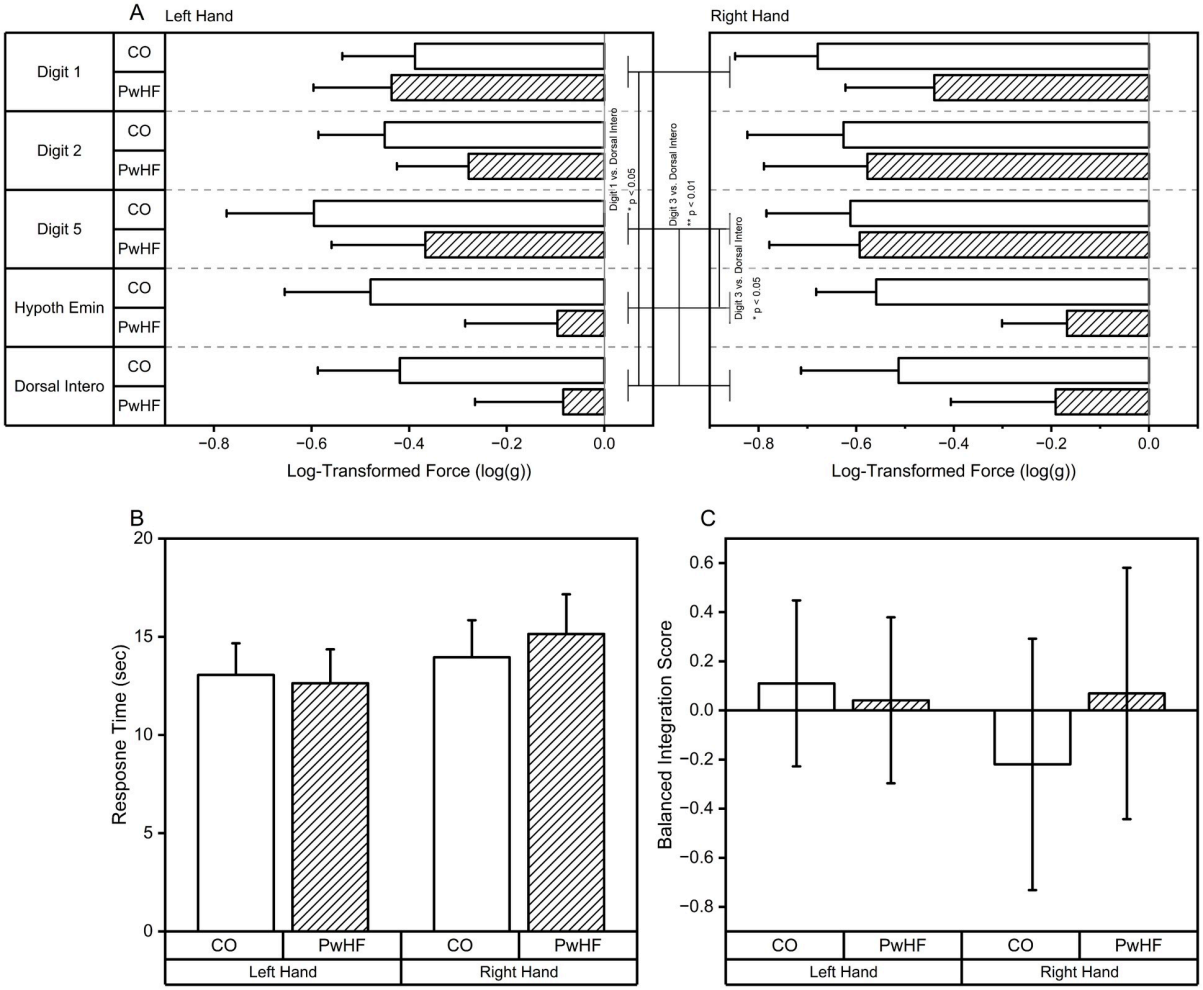
Hand sensory function data. A: Tactile sensory function data for both groups and all five tested sites (Digit 1, Digit 2, Digit 5, Hypothenar eminence (Hypoth. Emin.), and 1^st^ dorsal interosseus (Dorsal Intero.). B: Response time in stereognosis for both groups and both hands. C: Balanced integration score in stereognosis for both groups and both hands.

ALM identified SBP, HbA_1c_, DBP, heart failure duration, and SPO2 as covariates. Three-way MD-ANCOVA was performed, the main effect of *Site* remained significant (F_4,48_ = 2.65, p < 0.05, η^2^ = 0.18) (Fig 4A). The follow-up analysis of the main effect of *Site* demonstrated that 1^st^ dorsal interosseus had a higher tactile threshold compared to digits 1 (p < 0.05) and 5 (p < 0.01). Additionally, the follow-up analysis revealed that the hypothenar eminence had a higher tactile threshold compared to digit 3 (p < 0.05). SBP was significantly related to the threshold of tactile sensitivity (SBP: F_1,12_ = 7.02, p < 0.05). Threshold of tactile registration was positively correlated with SBP, HbA_1c_, DBP, and heart failure duration (SBP: r_200_ = 0.36, p < 0.001, HbA_1c_: r_190_ = 0.39, p < 0.001, DBP: r_200_ = 0.15, p < 0.05, heart failure duration: r_200_ = 0.18, p < 0.01), and was negatively correlated with SPO2 (r_200_ = -0.15, p < 0.05).

### Stereognosis

There was a group difference between CO and PwHF in stereognosis. PwHF took longer to identify the combination of numbers on dominos compared to CO, while the response accuracy was comparable between groups. The group difference remained significant in balanced integration score. When covariates were controlled, the heart failure duration became significantly related to response time and balanced integration score while the main effect of *Group* was removed (Fig 4B and 4C).

PwHF took longer to identify the numbers on dominos regardless of hand used (CO vs. PwHF (mean ± SE): 11.30 ± 1.63 vs. 16.17 ± 1.63). MD-ANOVA revealed a significant main effect of *Group* (F_1,18_ = 4.52, p < 0,05, η^2^ = 0.20). ALM identified heart failure duration, BMI, and HbA_1c_ as covariates for response time data. Controlling covariates removed the significant main effect of GROUP (Fig 4B). MD-ANCOVA revealed that heart failure duration was significantly related to response time (F_1,14_ = 4.82, p < 0.05, η^2^ = 0.26, with r_40_ = 0.57, p < 0.001).

Analysis of response accuracy data indicated that the accuracy of identifying the dominos is comparable between PwHF and CO. ALM identified HDL, SBP, and DBP as covariates for response accuracy. MD-ANCOVA did not find any significant main effects and significant interactions while controlling for covariates. DBP and HDL were negatively and positively correlated with response accuracy, respectively (DBP: r_40_ = -0.48, p < 0.01; HDL: r_38_ = 0.45, p < 0.01). SBP was not significantly correlated with response accuracy.

With respect to balanced integration score, MD-ANOVA indicated a significant main effect of *Group* (F_1,18_ = 4.74, p< 0.05, η^2^ = 0.21), such that PwHF exhibited lower balanced integration score compared to CO (CO vs. PwHF (mean ± SE): 0.53 ± 0.34 vs. -0.53 ± 0.34). ALM identified heart failure duration, DBP, and MoCA as covariates of balanced integration score. MD-ANOCVA found neither significant main effects or interactions (Fig 4C); however, heart failure duration and DBP were significantly related to balanced integration score (heart failure duration: F_1,15_ = 15.01, p < 0.001, with r_40_ = -0.54, p < 0.001; DBP: F_1,15_ = 14.82, p < 0.01, with r_40_ = -0.38, p < 0.05).

### Hand motor function

**Fig 5:**
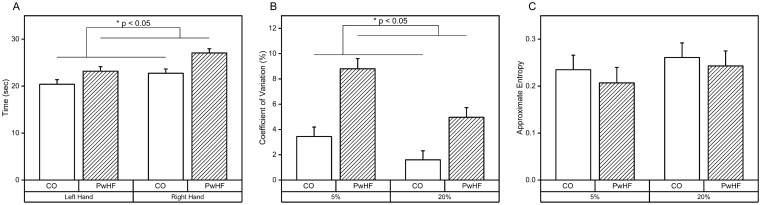
Hand motor function data. A: Group nine-hole peg test data for both groups and both hands. B: Group pinch force variability (coefficient of variation) data for both groups and both target force conditions. C: Group pinch force production regularity (ApEn) for both groups and both target force conditions.

Analysis of nine-hole peg test revealed that PwHF took longer to complete nine-hole peg test compared to CO regardless of which hand performed the task (CO vs. HF (mean ± SE): 21.69 ± 0.85 vs. 25.00 ± 0.85). MD-ANOVA found significant main effects of *Group* (F_1,18_ = 7.57, p < 0.05, η^2^ = 0.30) and *Hand* (F_1,18_ = 15.64, p < 0.001, η^2^ = 0.47). Across both *Groups*, nine-hole peg test took longer to complete with the left hand as compared to the right hand (right hand vs. left hand (mean ± SE): 21.79 ± 0.62 vs. 24.90 ± 0.81). ALM identified SpO2, MoCA, and BMI as covariates. The main effect of *Group* remained significant (F_1,14_ = 5.47, p < 0.05, η^2^ = 0.40) while controlling for the effect of SpO2, MoCA, and BMI (Fig 5A). Covariates MoCA and SpO2 were significantly related to the time to complete the nine-hole peg test (MoCA: F_1,15_ = 5.60, p < 0.05, η^2^ = 0.27 with r_40_ = -0.44, p < 0.01; SpO2: F_1,15_ = 12.52, p < 0.01, η^2^ = 0.46).

Analysis of pinch force variability (CV) during the constant pinch force tracking test revealed that CV was greater in the 5% target force condition and was comparable between CO and PwHF (5% vs. 20% (mean ± SE): 6.08 ± 0.76 vs. 3.31 ± 0.49). MD-ANOVA indicated a significant main effect of *Target* (F_1,18_ = 17.74, p < 0.01, η^2^ = 0.50). ALM identified HbA_1c_, heart failure duration, and DBP as covariates. MD-ANCOVA showed a main effect *Group* (F_1,14_ = 21.90, p < 0.001, η^2^ = 0.61) while controlling HbA_1c_, duration of heart failure diagnosis, and DBP (CO vs. HF (mean ± SE): 2.52 ± 0.55 vs. 6.89 ± 0.59) (Fig 5B). The identified covariates were significantly associated with CV (HbA_1c_: F_1,14_ = 24.49, p < 0.001, η^2^ = 0.64, heart failure duration: F_1,14_ = 18.55, p < 0.001, η^2^ = 0.57, DBP: F_1,14_ = 10.73, p < 0.01, η^2^ = 0.43). CV was positively correlated with HbA_1c_ (r_38_ = 0.44, p < 0.01).

Pinch force error from the target force (RMSE) were not different between groups (HF vs. CO) nor target conditions (5% vs. 20%). MD-AVNOA found neither main effects of *Group*, *Target n*or *Group* × *Target* interactions. No covariates were identified by ALM.

ApEn of pinch force did not exhibit *Group* differences but did exhibit differences due to *Target* pinch force; the 20% of target force condition had a higher ApEn value compared to 5% of target force condition (5% vs. 20% conditions (mean ± SE): 0.22 ± 0.02 vs. 0.25 ± 0.02), confirmed by MD-ANOVA with *Target* (F_1,18_ = 5.72, p < 0.05, η^2^ = 0.24). ALM identified HDL and HbA_1c_ as covariates of ApEn. MD-ANCOVA revealed that HDL was significantly related to ApEn (HDL: F_1,15_ = 4.59, p < 0.05, η^2^ = 0.23, with r_38_ = -0.35, p < 0.05) with no main effects or interactions (Fig 5C).

## Discussion

The purpose of this study was to examine hand sensorimotor function in PwHF. The subjective measurements of functional independence did not support our first hypothesis as the scores of ADL and IALDs were comparable between groups. The result of nine-hole peg test supports our second hypothesis given that PwHF took longer to complete the task compared to CO. Our third hypothesis was partially supported. Although the threshold of tactile registration was comparable between the two groups, PwHF exhibited slower response time and lower balanced integration score compared to CO in stereognosis. Although the group difference was removed when covariates for stereognosis were controlled, the heart failure duration was associated with the response time and balanced integration score. Finally, the assessments of constant pinch force tracking test revealed that the ability to maintain the pinch force at a constant level is decreased in PwHF. Together, these results suggest that heart failure leads to hand sensorimotor function impairment, which precedes declining independence in self-care daily tasks. In the following paragraphs, we will discuss potential underlying mechanisms of reduced hand sensorimotor function.

### Comparable functional independence

Analysis of functional independence scores revealed that PwHF in our study are independent in performing daily self-care tasks, which is not in line with clinical epidemiology investigations examining the functional independence in PwHF [6–10]. Multiple factors are involved in declined functional independence in PwHF [4]. Numerous studies report that cognitive impairment is a significant factor that contributes to the declined self-care functionality in PwHF [11, 44]. In our study, the total score of MoCA in PwHF ranged from 21 to 28; however, the scores of ADLs and IADLs did not vary. These results suggest that decline in cognitive function may not be the primary cause of declined functional independence in PwHF, but rather one of the multiple factors that lead to decline in functional independence. For instance, studies have reported that the combination of cognitive impairment and multi-morbidity explains the declined ADLs in PwHF [10]. These reports suggest that managing factors that contribute to declined self-care functionality is critical.

### Impaired multisensory information processing without a decline in tactile registration threshold

The battery of hand sensory function assessments revealed that PwHF have a reduced ability to integrate tactile and proprioceptive information to recognize objects while tactile sensitivity is at a normal level. These results indicate that the repercussions of heart failure on sensory function are not necessarily at the peripheral level (sensory mechanoreceptor), but rather at the central level.

Analysis of stereognosis suggested that heart failure has negative impacts on the ability to identify a handheld object using both tactile and proprioceptive information. PwHF exhibited longer response time while demonstrating comparable response accuracy compared to healthy control participants. As one person living with heart failure might use a different strategy (accuracy over speed) compared to other PwHF (speed over accuracy), we calculated balanced integration score to take the speed-accuracy tradeoff into account. PwHF had a lower balanced integration score value compared to control participants, suggesting that the negative effect of heart failure on stereognosis is true regardless of the strategy used by PwHF. Given that PwHF had a normal level of tactile function, the underlying mechanism of declined stereognosis may be due to slower multisensory information processing speed. Indeed, PwHF had a lower score in PCPST compared to CO, highlighting a decline in sensory information processing speed. This finding is in line with a study reporting reaction time deficits in approximately 40% of PwHF [45].

### Reduced fine motor skills and ability to maintain submaximal pinch force

Despite comparable self-care functionality between groups, the study found that fine motor skills are declined in PwHF compared to CO. Furthermore, pinch force steadiness was lower in PwHF, which may directly relate to prolonged motor task performance [17]. For example, PwHF took longer to complete the nine-hole peg test compared to CO regardless of the hand used, which is in line with reports of increased motor performance time in persons living with end-stage cardiac disease [46]. Together, hand motor function assessment may be a useful tool to detect motor impairment before PwHF notice challenges in their daily tasks.

Fine motor skills are associated with both peripheral and central factors. The tactile spatial acuity of a fingertip is typically associated with timed tests of fine motor skills, such that lower tactile spatial acuity coincides with a longer time to complete timed tests of fine motor skills [14]. As PwHF had a normal level of tactile registration threshold, it is unlikely that peripheral factors contributed to the declined fine motor skills in PwHF in this study. An alternative cause of declined fine motor skills may be declined force steadiness [17, 18]. For instance, 36% of the time to complete a motor task was accounted for by the ability to maintain 5% of the maximal voluntary contraction in manual tasks [17]. Similarly, approximately 60% of the variance in manual motor tasks has been explained by age and CV [18]. Given that PwHF exhibited comparable hand muscle strength and reduced force steadiness (higher CV) compared to age-matched healthy control participants, a greater pinch force fluctuation may be an underlying cause of declined fine motor skills in PwHF.

### Neurovascular disease and sensorimotor function

The evidence base demonstrates structural and functional abnormalities of the nervous system concurrent with vascular disease [47, 48]. Our study contributes by demonstrating reduced sensory and motor function of hands in PwHF. Given the findings in our study, is likely that the source of these deficits resides in the central nervous system.

Neuroimaging studies have demonstrated diffuse structural abnormalities of the brain in PwHF [4]. These structural abnormalities of the brain in PwHF seem to be caused by multiple factors, including cerebral hypoperfusion, oxidative stress, systematic inflammation, blood-brain barrier damages [48–50]. While left ventricular function seems to be the primary cause of structural abnormalities of the brain, heart failure-related impairment in neurovascular coupling may also contribute to cortical abnormalities [47, 51].

These structural abnormalities are likely the primary driver of sensory and motor function impairments observed in the current study. Our data suggest that PwHF have slower multisensory information processing and integration, which may be due to impairments in high-order processing of sensory information rather than early sensory processing. Multisensory integration requires cognitive control, which is reduced in PwHF [52]. As PwHF exhibited prolonged P300 latency, an index of information processing speed, the slow multisensory information processing may be attributed to the slower sensory processing speed [53–55].

This is in line with studies reporting deficits in PwHF in high-order cognitive domains, such as attention and psychomotor speed [56, 57]. Structural abnormalities in PwHF are also seen in prefrontal cortex, a cortical region responsible in the high-order processing [58–61]. Recent neuroimaging work has also reported that an oxygenated hemoglobin in prefrontal cortex is reduced in PwFH relative to healthy individuals [62]. Collectively, heart failure-related structural abnormalities of the brain may lead to reduced cortical activity in the prefrontal cortex, which in turn, affects the ability to process multisensory information.

Impaired prefrontal cortex function is likely coupled with impaired cognitive function (specifically attention) in PwHF [63]. Attention is a limited cognitive resource used to process sensory information in voluntary movement [64]. Declined attentional resources and greater needs of attention in voluntary movements may contribute to motor impairments in persons living with cognitive impairments. Studies have demonstrated reduced motor function in person living with cognitive impairment [65]. For instance, a dual-tasking paradigm, in which participants perform motor and cognitive tasks simultaneously, greater dual-tasking interference existed on either or both motor and cognitive tasks in person living with cognitive impairments; with exaggeration of deficits increasing as the difficulty of dual tasking increased [66, 67]. Voluntary movements may also be more cognitively demanding in PwHF due to functional reorganization of the brain associated to the structural abnormalities [68, 69]. Together, the reduced motor function of the hands in PwHF may be due to changes in attentional resources and its role in movement control.

Changes in motor neuron inputs may also be a plausible explanation for reduced motor function in PwHF. Force steadiness research has demonstrated that mechanisms of fluctuation in force steadiness are multifactorial; however, the neurophysiological mechanism that leads to a greater force fluctuation is variability in motor unit activity [70]. Although remodeling of motor units was initially thought as the cause of reduced force steadiness, recent evidence indicates that common input to motor neurons may instead be the cause of force fluctuation [71–74]. The origin of common input to motor neurons is still unclear; however, cortical structural abnormalities in the upper stream of corticospinal tract might increase fluctuation in common synaptic input [18, 75]. Given the structural abnormalities of the brain in PwHF, the motor neuron inputs may be different from their counterparts group. Future studies should examine whether motor neuron inputs change by heart failure.

### Open questions in sensorimotor function in heart failure

#### Complications of heart failure risk factors

We used ALM to identify covariates for the hand sensorimotor data; the duration of heart failure, DBP, and HbA_1c_ were the covariates that were associated with hand sensorimotor function assessments, including Semmes-Weinstein Monofilament test, Stereognosis, and Constant Pinch Force Tracking test. Such observation indicates that the declined hand sensorimotor function we observed in PwHF may not be purely due to heart failure, but the combination of heart failure and other comorbidities, such as type II diabetes mellitus (T2DM) and arterial hypertension. Both T2DM and arterial hypertension, which are known for risk factors of heart failure, affect sensory and motor function [27, 76–78]. Complications that contributes to sensorimotor impairments in T2DM and arterial hypertension is peripheral neuropathy include peripheral neuropathy, which is characterized by distal-to-proximal losses of peripheral nerve fibers as a result of metabolic and microvascular alterations and chronic hyperglycemia, and cognitive impairment [79]. The effect of peripheral neuropathy on sensorimotor function depends on which never was damaged since the injury to the nerve ranges from small and large sensory nerves to motor nerves. Smaller sensory nerves, sensory nerves that convey pain-, temperature-, and tactile-related signals, are affected at the early stage of peripheral neuropathy, whereas the larger sensory nerves, sensory nerves that convey proprioceptive signals, are affected later [80]. Disrupted tactile and proprioceptive signals can lead to motor impairments as sensory information is critical in controlling movements [67, 81]. Future investigations examining sensory and motor function in PwHF should perform neuropathy assessment via nerve conduction examination, for example, to consider its impact on sensory and motor nerves. Such assessment will help investigators to deepen their understanding in sensory and motor function impairments in PwHF.

Similar to PwHF, persons living with T2DM and arterial hypertension exhibit structural and functional abnormalities of the brain, in which can contribute to the sensory and motor function impairment [82–84]. While evidence demonstrating the structural abnormalities of the brain in PwHF is growing, there is very limited studies using neuroimaging technique, such as functional magnetic resonance imaging, electroencephalography, and functional near-infrared spectroscopy, to assess functional abnormalities of the brain during a sensory and motor tasks in PwHF [4]. To elucidate the cortical mechanisms of sensory and motor impairments in PwHF, future investigations should employ neuroimaging techniques. The employment of neuroimaging technique in PwHF valuable as discovering cortical areas involved in sensorimotor function impairments can serve as a framework for neurorehabilitation as the cortical areas can be served as cortical target for non-invasive brain stimulation, such as repetitive transcranial magnetic stimulation, transcranial direct current stimulation, and transcranial ultrasound stimulation [85–87].

## Limitations

The study is limited by the small sample size; therefore, our results may not be generalized. Heart failure is a heterogenous health condition since there are no definitive mechanisms that cause heart failure. The study attempted to collect variables to characterize heart failure in our study by collecting duration of heart failure diagnosis, self-reported ejection fraction, self-reported subtypes of heart failure, and the total walking distance of six-minute walk test; however, ejection fraction and subtypes of heart failure were incomplete. Additionally, our study is lacking other variables that could be helpful to characterize PwHF, such as echocardiogram and other biomarkers specific to heart failure. Because of this, the results reported in this study are not sufficient to examine whether hand sensorimotor function differs between subtypes of heart failure (e.g. HFrEF vs, HFpEF) or the classes of New York Heart Association. Additionally, current study is lacking assessments of neurophysiology (e.g. nerve conduction velocity and corticospinal excitability), neuroimaging technique for assessing the cortical activity during the task, and assessments of heart health (e.g. echocardiogram and electrocardiogram). Such limits prevent a deeper understanding the relationship between heart failure and sensory/motor function of hands. To overcome these limitations, multi-site (i.e. different locations), multi-institutional (i.e. clinical and research institutions), and multi-disciplinary (i.e. neuroscience and cardiology) examination is necessary [88].

## Conclusion

Little attention is given to sensorimotor dysfunction in PwHF, despite complaints of declined self-care functionality [4]. Given the adverse outcomes of declined ADLs in PwHF, the need to understand the cause of reduced ADLs is higher than ever. The current study demonstrated that heart failure has repercussions on hand sensorimotor function. Critically, PwHF did not report difficulty in performing daily tasks. The cause of reduced hand sensorimotor function likely resides in the central nervous system, including cognitive impairment and variability in motor unit activity. This study helps build better understanding of declined functional care capabilities in PwHF. To further understand the underlying mechanisms of reduced hand sensorimotor function in PwHF, discover potential intervention targets, and determine whether assessments of hand sensorimotor function can serve as a vehicle to quantify restoration of self-care functionality, multidisciplinary and multisite collaboration is crucial in future work.

## Supporting information

S1 Table Medication list. List of medications reported by PwHF. PwHF stands for persons living with heart failure, NA stands for not applicable.
(DOCX)
