# Supplementary material for: Preliminary report: Reduced hand sensory and motor function in persons living with heart failure
Source: PLoS One. 2024 Nov 15;19(11):e0312468. doi: 10.1371/journal.pone.0312468 (PMC11567519; doi:10.1371/journal.pone.0312468)
Supplement: S1 Table — List of medications reported by PwHF. PwHF stands for persons living with heart failure, NA stands for not applicable. (DOCX) [file pone.0312468.s001.docx]

| Participant | List of medication reported by each participant |
| --- | --- |
| PwHF 1 | NA |
| PwHF 2 | Entresto, Aspirin, Carvedilol |
| PwHF 3 | Sotalol, Atorvastatin, Losartan, Xarelto, Aspirin |
| PwHF 4 | NA |
| PwHF 5 | NA |
| PwHF 6 | Hydrochlorothiazide, Eliquis, Risperidone, Buspar, Metformin |
| PwHF 7 | NA |
| PwHF 8 | Metoprolol, Amiodarone, Calcium Acetate, Warfarin, Zolpidem |
| PwHF 9 | Amlodipine, Entresto, Labetalol, Torsemide, Acetylsalicylic Acid, Ultram |
| PwHF 10 | Atorvastatin, Losartan, Metoprolol, Amlodipine, Furosemide |

**S1 Table: Medication list.** List of medications reported by PwHF. PwHF stands for persons living with heart failure, NA stands for not applicable.
